# Quantitative Analysis of the Transcriptome of Two Commonly Used Human Monocytic Cell Lines—THP-1 and Mono Mac 6—Reveals Their Arrest during Early Monocyte/Neutrophil Differentiation

**DOI:** 10.3390/ijms23105818

**Published:** 2022-05-22

**Authors:** Srinivas Akula, Sandra Lara, Anna-Karin Olsson, Lars Hellman

**Affiliations:** 1 Department of Cell and Molecular Biology, Uppsala University, The Biomedical Center, P.O. Box 596, SE-75124 Uppsala, Sweden; srinivas.akula@slu.se (S.A.); slaramart@gmail.com (S.L.); 2 Department of Anatomy, Physiology, and Biochemistry, Swedish University of Agricultural Sciences, Box 7011, SE-75007 Uppsala, Sweden; 3 Department of Medical Biochemistry and Microbiology, BMC, Box 582, SE-75123 Uppsala, Sweden; anna-karin.olsson@imbim.uu.se

**Keywords:** monocyte, neutrophil, cell line, THP-1, Mono Mac 6, in vitro

## Abstract

Cell lines of monocyte/macrophage origin are often used as model systems to study monocyte/macrophage biology. A relevant question is how similar these cell lines are to their in vivo counterparts? To address this issue, we performed a detailed analysis of the transcriptome of two commonly used human monocyte/macrophage cell lines, Mono Mac 6 and THP-1. Both of these cell lines originate from leukemic cells with myelo-monocytic characteristics. We found that both Mono Mac 6 and THP-1 represent cells of very immature origin. Their transcriptomes show more similarities to immature neutrophils than cells of the monocyte/macrophage lineage. They express significant levels of N-elastase, proteinase 3, cathepsin G, and azurocidin but very low levels of CD14, ficolin, and complement factor P. All major MHC class II genes are also expressed at low levels. They show high levels of lysozyme and low levels of one of the immunoglobulin Fc receptors, FCGRIIA, which is characteristic of both neutrophils and monocytes. THP-1, but not Mono Mac 6, also expresses the high-affinity receptor for IgG, FCGRIA. Both cell lines lack the expression of the connective tissue components fibronectin, proteoglycan 4, and syndecan 3, which are characteristics of tissue macrophages but are absent in blood monocytes, indicating that they originate from bone marrow precursors and not yolk sac-derived hematopoietic cells. Both of these cell lines seem, therefore, to represent cells arrested during early myelo-monocytic development, at a branch point between neutrophil and monocyte differentiation. Their very immature phenotype indicates that great care should be taken when using these cell lines as models for normal monocyte/macrophage biology.

## 1. Introduction

Cell lines have been instrumental for many areas of cell biology and also an essential tool for the production of properly folded mammalian proteins for structural and functional analysis as well as for therapeutic use. Cell lines often originate from tumor cells selected for in vitro growth since their normal counterparts are often short lived and change phenotype quickly after being put in culture. Cell lines originating from different hematopoietic lineages have been of major importance for the analysis of signaling pathways regulating cell activation and development. However, the question is always, how similar are they to their normal in vivo counterparts?

The two most frequently used in vitro models for studies of human monocytes/macrophage biology are the cell lines Mono Mac 6 and THP-1. Both of them originate from leukemias with myelo-monocytic characteristics. Mono Mac 6 is derived from the peripheral blood of a 64-year-old male with acute monocytic leukemia (AMoL), while THP-1 comes from the peripheral blood of a 1-year-old boy with AMoL, both with monocytic features [1,2]. These cell lines have been used by many labs, and the number of publications including them can now be counted in many hundreds or even thousands. A search in PubMed for publications involving Mono Mac 6 resulted in 351 hits and as many as 14,560 hits for THP-1, indicating their high impact in various studies of biological processes involved in monocyte/macrophage biology and also for more general cell biology.

Almost 30 years ago, we performed a Northern blot analysis of 18 different marker genes in Mono Mac 6, THP-1, and a few other hematopoietic cell lines [3]. This study indicated that Mono Mac 6 and THP-1 were arrested at a very early stage of myelo-monocytic development. However, the number of markers analyzed was relatively limited. The rapid progress in different high-throughput technologies for whole-genome transcriptome analysis has now made it possible to extend this early study to include all 21,000 human genes to obtain a more complete picture of the phenotype of these cell lines. Such a detailed analysis can serve as a guide for their use as in vitro models of human monocyte/macrophage biology, but also as models of human myelo-monocytic leukemias.

Human monocytes and neutrophils have been found to share a common bipotential progenitor [4,5,6]. As previously indicated from the Northern blot analysis, both Mono Mac 6 and THP-1 appear to represent cells arrested at or close to this bipotential branchpoint between monocyte and neutrophil development. We can now increase the details of this analysis to show that these two cell lines express several of the major granule components of human neutrophils, including N-elastase, proteinase 3, cathepsin G, myeloperoxidase (MPO), defensin and azurocidin. They also express lysozyme and FCGRIIA, which are markers for both monocytes and neutrophils. However, they almost completely lack expression of CD14, CD4, properdin, and ficolin, of which all four are important markers for human monocytes. They also lack the expression of several connective tissue components that are markers for tissue macrophages, indicating that these cell lines originate from bone marrow hematopoietic cells and not from yolk sac-derived cells. Yolk sac-derived cells are the precursors of the absolute majority of all tissue macrophages [7,8,9,10]. The very immature phenotype of these cells and that they show more similarities to immature neutrophils than monocytes indicate that great care should be taken when using these cell lines as models for normal monocyte/macrophage biology.

## 2. Results

### 2.1. Cells, RNA Isolation and Transcriptome Analysis

After expansion in cell culture medium containing 10% fetal calf serum, approximately 4 million cells of both Mono Mac 6 and THP-1 were pelleted and washed once with PBS before preparation of total RNA. The RNA was then sent to SciLife lab NGI facility in Uppsala for Ampliseq analysis of their total transcriptome. The data were delivered in the format of the number of reads for each gene in an Excel file. This Excel file was then analyzed manually for the most relevant marker genes to determine the stage of arrest in the development of these two cell lines. As reference material, we also added the reads from two freshly isolated samples of human peripheral blood monocytes and one pooled sample of peritoneal macrophages isolated from 30 Balb/c mice. This reference material was collected from two of our recent publications [11,12]. In the monocyte article, there is information from five separate individuals [12]. Two representative samples from this analysis are shown in Table 1. Two individual samples from the Mono Mac 6 culture were also analyzed separately to show the high reproducibility of this type of analysis (Table 1). These two samples are shown as Mono Mac 6:1 and Mono Mac 6:2 in Table 1.

### 2.2. Expression Levels of Monocyte/Macrophage Marker Genes

Both Mono Mac 6 and THP-1 originate from patients with monocytic leukemias. We therefore started to look for marker genes of special interest for monocyte/macrophage biology such as CD14, lysozyme, CD4, a number of immunoglobulin Fc receptors, and a few complement components that we recently observed being expressed at relatively high levels in freshly isolated human blood monocytes such as properdin and ficolin [11,12]. Lysozyme levels were relatively high in both cell lines, with the highest levels in Mono Mac 6, which had approximately 50% of the levels observed in blood monocytes (Table 1A). In THP-1, the levels were approximately 5% of what was seen in blood monocytes (Table 1A). In contrast, CD14 was almost undetectable in THP-1, with only 7 reads, and very low levels in Mono Mac 6 with levels in the range of 40–50 reads. CD4 was totally absent in Mono Mac 6 and relatively low in THP-1 with 182 reads (Table 1A). Both properdin and ficolin were almost undetectable in both cell lines (Table 1A). No or very low expression levels of CD40 of CD86 (also named B7:2) and of MARCO, a macrophage marker, were observed in both cell lines (Table 1A).

Both cell lines were also negative for the majority of connective tissue components that previously have been found to be highly expressed in peritoneal macrophages, including fibronectin, proteoglycan 4, syndecan 3, and extracellular matrix protein 1 (Table 1A). It was only extracellular matrix protein 1 that was expressed at a significant level in one of the cell lines, in THP-1, with 211 reads (Table 1A). The core protein for the granule stored proteoglycans, the serglycin, was expressed at high levels in all the cells tested, including the peritoneal macrophages, the human blood monocytes, and both cell lines (Table 1A). Serglycin is in monocytes and macrophages the core protein for synthesis of granule stored chondroitin sulfate.

Of the Fc receptors, it was primarily the FCGR2A that was expressed in both cell lines at significant but very low levels, Mono Mac 6 with 66 and 70 reads and THP-1 with 80 reads (Table 1B). The level of this receptor in normal human monocytes is 10–20-fold higher than in Mono Mac 6 and THP-1, in the range between 500 and 1200 reads (Table 1B). Human monocytes also express FCGRIIIA with levels of 100–300 reads, a receptor that was almost undetectable in both Mono Mac 6 and THP-1 (Table 1B). The high-affinity IgG receptor, FCGR1A, was found to be expressed by THP-1 with 203 reads. Mono Mac 6 showed, in contrast, very low levels of this receptor, only around 20 reads (Table 1B). Relatively low levels of this high-affinity receptor were also found on the blood monocytes with 51 and 60 reads in the two individuals analyzed (Table 1B). Concerning other immunoglobulin Fc receptors, both Mono Mac 6 and THP-1 lack expression of the majority of the IgG receptors, including FCGRIIIA, FCGRIIIB, FCGRIIB, and both the high- and the low-affinity receptors for IgE (FCERIA and FCERII) as well as the IgA, IgM, and IgA + IgM receptors (FCARI, FCAMR, and FCMR) (Table 1B). The only component that is high or relatively high in both cell lines and also in human monocytes is the FCER1G (Fc epsilon receptor gamma chain), which is part of both the IgE and several of the IgG receptors.

Regarding the major histocompatibility genes, we found that Mono Mac 6 has relatively high levels of all three MHC class I alpha chains, the HLA-A, HLA-B, and HLA-C, with 500–750 reads, whereas THP-1 almost completely lacks MHC class I expression in spite of that both cell lines express high levels of the beta chain, the b2-microglobulin (B2M) (Table 1C). Both cell lines also almost completely lack expression of the class II genes, except for the expression of one beta chain in THP-1 (DRB1) with 303 reads and only 35 and 37 reads in Mono Mac 6 (Table 1C). A low level of DRA was also seen in Mono Mac 6 with 61 and 69 reads. This should be compared with human blood monocytes with expression levels from 3500 to 5500 reads, or approximately 75 times higher expression levels (Table 1C).

We also observed quite varying expression levels between the two cell lines with respect to several of the enzymes involved in the production of leukotrienes and prostaglandins. No expression was observed for arachinodate 15-lipoxygenase (ALOX15), phospholipase A2 (PLA2G7), or arachinodate 5-lipoxygenase (ALOX5), but very high levels of arachinodate 5-lipoxygenase activating protein (ALOX5AP) in Mono Mac 6, with over 500 reads and very low level in THP-1 with 149 reads (Table 1D). Mono Mac 6 also expressed low levels of prostaglandin I synthase (PTGIS) with 90 and 105 reads, whereas this transcript was absent in THP-1 (Table 1D).

A low level of apolipoprotein E (APOE) was observed in both cell lines, with expression levels in the range of 50 to 60 reads (Table 1D). Low levels of the oxygen radical-forming enzyme of the cytochrome b245 or b558 involved in bacterial killing were observed in Mono Mac 6 with 159 and 232 reads compared to the very low level in THP-1 with only 19 reads. High levels of this transcript were seen in the human monocytes with 867 and 1081 reads and even higher in the mouse macrophages with 2664 reads (Table 1E).

### 2.3. Expression Levels of Marker Genes Characteristic of Neutrophilic Granulocytes

Relatively high levels of several neutrophil-related transcripts such as N-elastase, cathepsin G, azurocidin, and proteinase 3 were detected in both of these cell lines (Table 1F). They both expressed high levels of N-elastase with over 2500 reads, and for azurocidin, 2410 reads in THP-1. Mono Mac 6 expressed slightly lower levels in the range of 1700 and 900 for N-elastase and azurocidin, respectively (Table 1F). The two cell lines differed markedly in the expression of proteinase 3. THP-1 had a very high level of proteinase 3 with 2635 reads, whereas Mono Mac 6 only had relatively low levels of this neutrophil serine protease with approximately 65 reads (Table 1F). Cathepsin G was instead the dominating neutrophil protein expressed in Mono Mac 6 with approximately 7700 reads. THP-1 did also express high levels of this serine protease with 1868 reads (Table 1F). We also detected low levels of myeloperoxidase (MPO) and BPI in freshly isolated human monocytes with 6 and 18 reads for MPO and 16 and 62 for BPI in the two donors (Table 1F).

When looking at other neutrophil-specific markers such as lactoferrin, bacterial permeability-increasing protein (BPI), cathelicidin, and defensin, we could see that both cell lines lacked expression of lactoferrin and cathelicidin. Low levels of BPI were detected in Mono Mac 6 with 16 reads, and a low level of defensin was observed in THP-1 with 60 reads (Table 1F).

### 2.4. Expression of Marker Genes Characteristic of Other Hematopoietic Lineages

One of these cell lines, Mono Mac 6, but not THP-1, expressed low levels of the mast cell and basophil-specific marker tryptase beta-2, with 179 and 197 reads in the two donors (Table 1F). We also looked for the expression of eosinophil-related marker genes. We did not detect any expression of two of the eosinophil-related proteins, the major basic protein MBP and eosinophil peroxidase (EPO), but low levels of eosinophil cationic protein (ECP) in Mono Mac 6 with 123 and 128 reads and high levels of eosinophil-derived neurotoxin (EDN) in both cell lines with 786, 798, and 1076 reads, respectively (Table 1F).

## 3. Discussion

Cell lines have been essential for the analysis of the majority of cellular processes and thereby for our understanding of basic cell biology. They have also been of major importance for our view of differentiation by defining the phenotype of mature and immature cells of different cell lineages. However, they are often arrested in the early stages of development, when proliferation still is high. Data from such cell lines, therefore, need to be taken with great care as they may differ markedly from their in vivo counterparts, which are often terminally differentiated.

Lymphoid cell lines have been a very important tool in the dissection of the different steps in the rearrangement of immunoglobulin and T-cell receptor genes and the enzymes involved in the rearrangement process. Another example is mast cell lines, which have been instrumental in defining the various components of the high-affinity IgE receptor and the signaling molecules involved in mast cell activation and granule release, as well as the cloning of the majority of granule components. Similarly, the human promyelocytic cell line HL-60, isolated from the peripheral blood of a 36-year-old female, has been a very important tool for the cloning of the majority of neutrophil granule components [13]. Mature human neutrophils are difficult to use for this purpose since transcription of granule component genes is almost completely turned off just before exit from the bone marrow. However, when using these cell lines as models for normal cellular functions, it is essential to know their phenotype and characteristics. A detailed quantitative analysis of their transcriptome can therefore serve as a guide to their use in different experimental settings.

By a quantitative analysis of the two myelo-monocytic cell lines Mono Mac 6 and THP-1, we can now extend the earlier analysis of these cell lines performed by Northern blot to provide dramatically increased detail in the phenotype of these two cell lines. In agreement with the earlier analysis, both of these cell lines display a phenotype of very immature cells expressing both neutrophil and some monocytic markers indicating that they correspond to cells at the branch point between monocyte and neutrophil development. However, they both seem to have a bias toward neutrophil development. Both cell lines almost completely lack expression of MHC class II, an important characteristic of human blood monocytes, and they also lack the expression of essentially all connective tissue components, such as fibronectin, proteoglycan 4, and syndecan 3, which are characteristics of tissue macrophages, but absent in blood monocytes (Table 1A) [11,12]. In some aspects, Mono Mac 6 and THP-1 show characteristics that match the phenotype of human blood monocytes, such as the expression of lysozyme and FCGRIIA. However, the expression levels of this receptor for IgG were 10–20-fold lower than the expression in blood monocytes (Table 1B). The expression of the mast cell and basophil-specific protease, the beta-tryptase, in Mono Mac 6 shows that this cell line also has similarities to other myeloid lineages. Another characteristic that links this cell line to mast cells is the very high level of expression of cathepsin G. The expression of cathepsin G, which is absent in blood monocytes, was in the range of 7700 reads in Mono Mac 6 (Table 1F). Cathepsin G is the serine protease that shows the highest expression levels of all granule proteases in human skin mast cells [14]. However, cathepsin G is also one of the most abundant proteins in granules of human neutrophils [15]. Interestingly, expression of the mast cell tryptase has been observed in several myelo-monocytic cell lines and leukemias and is considered a potential marker in the diagnosis of myeloid leukemias [16].

**Figure 1:**
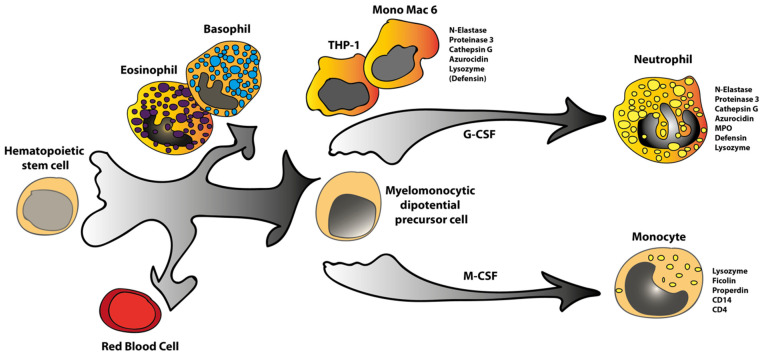
A schematic representation of the possible developmental arrest in differentiation of the cell lines THP-1 and Mono Mac 6. The tentative position during differentiation of THP-1 and Mono Mac 6 is depicted in the figure, in the context of the myeloid branch of human hematopoiesis, with specific emphasis on the later stages in myelomonocyte differentiation. Marker genes expressed by THP-1, Mono Mac 6, peripheral blood neutrophils, and peripheral blood monocytes are listed to the right of the pictures of the different cells.

The expression pattern of these two cell lines shows a very high degree of similarity in their transcriptomes, indicating that they originate from cells of a similar stage of differentiation at the branch point between monocyte and neutrophil development (Figure 1). However, there are also several differences, as described above. THP-1 expresses high levels of proteinase 3 but very low levels in Mono Mac 6. A low level of defensin b1 was detected in THP-1, but no expression of this anti-microbial peptide in Mono Mac 6. The mast cell tryptase was expressed in Mono Mac 6 but not in THP-1 (Table 1F). Except for these minor differences, the two cell lines are remarkably similar in their transcriptome. This is interesting in the light that they originate from persons of very different ages, 1 and 64 years of age. This fact may indicate the presence of a stage of differentiation that is particularly sensitive to oncogenic transformation. This indication would be interesting to follow up by analyzing a larger set of monocytic cell lines derived from monocytic leukemia patients. We have previously analyzed the HL-60 cell line for a limited set of transcripts by Northern blot [3]. We found that also this cell line shows many characteristics similar to THP-1 and Mono Mac 6. All three cell lines express N-elastase, cathepsin G, and proteinase 3 (myeloblastin) [3]. However, HL-60 also expresses high levels of MPO and defensin, indicating a more neutrophil-like phenotype compared to Mono Mac 6 and THP-1 [3].

The now increased detail in the analysis of the transcriptome of these two cell lines confirms the previous indications based on Northern blot analysis of a limited set of marker genes [3]. We can confirm that both THP-1 and Mono Mac 6 show very immature phenotypes with characteristics of cells at the branchpoint in differentiation between monocytes and neutrophil development (Figure 1). We will here stress the fact that this analysis is based entirely on transcriptome data. Concerning their use as cell line models for monocyte/macrophage development, our results indicate that both of these cell lines are relatively poor representatives as they show more similarities to neutrophils than monocytes. This needs to be taken into serious consideration when using these cell lines as models for monocyte biology. However, in favor of these cell lines are their relatively stable phenotype after almost 30 years, which is an indication that the risk of major changes in phenotype upon culturing is relatively limited.

## 4. Materials and Methods

### Cells, RNA Isolation and Transcriptome Analysis

THP-1 and Mono Mac 6 cells were maintained in RPMI1640 medium (Gibco Life Technologies, Carlsbad, CA, USA, 21875-034) supplemented with 10% fetal calf serum (Gibco Life Technologies, Carlsbad, CA, USA, 10500-064). The cells were expanded in standard culture flasks in the above medium without any addition of cytokines or other stimuli such as LPS and no serum starvation. Approximately 4 million cells were harvested and washed once with phosphate-buffered saline (standard PBS) before isolation of total RNA using the RNeasy Plus mini kit from (Qiagen, Hilden, Germany), according to the manufacturer’s recommendations. The RNA was eluted with 30 μL of DEPC-treated water, and the concentration of RNA was determined by using a Nanodrop ND-1000 (Nano Drop Technologies, Wilmington, DE, USA). Later the integrity of the RNA was confirmed by visualization on 1.2% agarose gel using ethidium bromide staining.

The THP-1 and Mono Mac 6 cells were analyzed for their total transcriptome by the Thermo-Fisher chip-based Ampliseq transcriptomic platform at the SciLife lab National Genomics Infrastructure (NGI) facility in Uppsala, Sweden (Ion-Torrent next-generation sequencing system, Thermo-Fisher, Waltham, MA, USA ). The sequence results were delivered in Excel files for an easy comparison of expression levels between samples.

Information from two previous studies of freshly isolated mouse peritoneal macrophages and human blood monocytes was included as reference material [11,12]. For the extraction of peritoneal cells, 30 mice were euthanized by neck dislocation during isoflurane anesthesia, the abdominal skin was removed, and 9 mL of ice-cold phosphate-buffered saline (PBS) was injected into the peritoneal cavity. After making sure that the injected PBS was thoroughly dispersed within the peritoneal cavity, peritoneal lavage fluid was collected, and the cells were pelleted by centrifugation at 400× *g* for 10 min. The cells were resuspended in PBS (pH 7.4) with 2% heat-inactivated fetal bovine serum (Gibco, Carlsbad, CA, USA), followed by incubation with the following fluorescent-labeled antibodies: F4/80 (BM8), CD11b (M1/70), CD19 (1D3), CD117 (2B8) and FcεRI (MAR-1). The antibodies were obtained from BD Biosciences (Franklin Lakes, NJ, USA) or eBioscience (Hatfield, U.K.). FACS-isolated peritoneal macrophages were collected for RNA isolation. The flow cytometry-based cell sorting was performed on a FACSAria III (BD Biosciences), and data were analyzed with FlowJo software (TreeStar Inc., Ashland, OR, USA).

The human peripheral blood monocytes were purified from buffy coats from the blood center at the Akademiska hospital in Uppsala. Peripheral blood mononuclear cells (PBMCs) were isolated using Ficoll–Paque Plus (GE Healthcare, Uppsala, Sweden) and standard density gradient centrifugation. PBMCs were further washed with PBS containing 2 mM of EDTA and incubated with anti-CD14-coated magnetic beads (Miltenyi Biotec, Bergisch Gladbach, Germany). Positive selection of CD14^+^ cells was performed through magnetic cell separation. Subsequently, CD14 cells were stained with antihuman CD14 PE antibody (clone: 61D3, Invitrogen, Carlsbad, CA, USA), and the purity was verified (average of 95%) by flow cytometry.

## Figures and Tables

**Table 1:** Expression levels for a panel of hematopoietic makers genes in mouse peritoneal macrophages, in two different human monocyte preparations (originating from two different donors) and from two human monocytic cell lines, Mono Mac 6 and THP-1. The number of reads corresponds to the expression level of these genes in the various cell preparations.

	Mouse Peritoneal MQ	H-Monoc. Male Age 51	H-Monoc. Female Age 61	Mono Mac 6:1	Mono Mac 6:2	THP-1
**A. Monocyte-related transcripts and housekeeping genes**						
CD14	627	1697	1525	43	53	7
CD4	0.6	594	1422	0	0	182
CD40	79	6	19	0.3	0.6	8
CD86 (B7:2)	257	236	108	9	5	0.2
MARCO	28	1	4	0.2	0	0
CD68 (Binds oxidized LDL)	638	1273	547	57	55	486
LYZ (Lysozyme)	105,000	27,394	16,400	8795	8366	1087
FCN1 (Ficolin A)	1306	3198	2183	0	0	0
CFP (Compl. factor P, Properdin)	5225	991	784	5	4	15
ACTB (beta actin)	7060	19,693	23,328	13,142	12,967	17,771
FN1 (Fibronectin)	25,920	0	0	0	0	0
PRG4 (Proteoglycan 4, Lubricin)	3606	0	0	0	0	0
SRGN (Serglycin)	2803	3855	5214	19,116	19,045	4522
SDC3 (Syndecan 3)	3205	5	7	0	0	20
ECM1 (Extracellular matrix prot.1)	3180	1	0.5	18	15	211
**B. Fc receptors**						
FCGR2A	-	580	1243	66	70	80
FCGR2B	9	59	87	0.3	0	1
FCGR3A (FCGR3)	1968	131	297	0	0	0.1
FCGR3B	-	22	6	0	0	0
FCGR1A	35	51	60	24	21	203
FCER1A	0	7	34	0	0	0.2
FCER2	0.3	2	3	0.4	0.4	0.3
FCER1G	1318	1173	2125	337	318	704
FCAR (CD89)	-	92	11	0	0	0.2
FCMR (FAIM3)	1	3	4	0.4	0.5	0
FCAMR	0	0	0	0	0	80
**C. MHC class I and II**						
HLA-A	-	1548	1755	634	750	98
HLA-B	-	6	1329	570	600	2
HLA-C	-	2979	1	554	706	4
B2M		5521	4642	1801	1536	2369
HLA-DRA	-	5490	3645	69	61	16
HLA-DRB1	-	3023	2188	37	35	303
HLA-DPA1	-	2375	4099	2	2	7
HLA-DPB1	-	1029	1506	4	5	3
**D. Lipid mediators and metabolism**						
ALOX15 (Arachinodate15 lipoxyg.)	12,680	0	0	0	0	0.1
PLA2G7 (Phosplipase A2)	1549	15	26	0.1	0.2	3
ALOX5AP (Arach.5-lipoxyg.act prot.)	1296	111	85	5170	5429	149
PTGIS (Prosaglandin I syntase)	1283	0	0	90	105	0
ALOX5 (Arach. 5 -lipoxygenase)	494	190	154	2	0.6	14
DPEP2 (Dipeptidase 2 memb. b.)	1243	77	85	0.5	0.5	0.4
APOE (Apolipoprotein E)	2413	2	4	52	65	51
PLTP (Phospholipidtransfer protein)	5788	1	1	76	101	54
**E. Anti-microbial and phagocytosis-related proteins**						
CYBB (Cytoch.b-245 (Nox2) Cytb558)	2664	1081	867	232	159	19
PADI4 (Peptidyl arg. deim. type IV)	1029	77	31	0	0	0
FLNB (Filamin B phagocytosis)	2235	9	9	103	152	53
FLNA (Filamin A)	1458	1175	494	866	1023	0
TIMD4 (Binds Phosph. Ser. apoptotic cells)	1244	0	0	0	0	0.1
**F. Neutrophil, eosinophil, and mast cell-related**						
MPO (Myeolperoxidase)	0	6	18	15	14	5
ELANE (Neutrophil Elastase)	0	0.7	1	1622	1817	2542
PRTN3 (Proteinase 3)	68	0	0	68	64	2635
CTSG (Cathepsin G)		0.1	0.8	7725	7712	1868
AZU1 (Azurocidin)	-	3	3	941	894	2410
LTF (Lactoferrin)	0.3	0	0.1	0	0	0
BPI (Bacterial permeability incr. pr.)	0	62	16	0	0	0
DEFB1 (Defensin beta 1)	0	0	0.2	5	4	60
Camp (Cathelicidin)	0	1	1	0	0.1	2
TPSB2 (Tryptase B2, MCs)		0	0.3	179	197	0.3
HDC (Histidine decarboxylase)	42	0	0.1	0	0	0
MRGPRX2 (MCs)	-	0	0	0	0	0
EPO (EPX)		0	0	0	0	0
ECP (RNASE3)		0	0.6	128	123	9
EDN (RNASE2)		14	40	786	798	1076
MBP (PRG2)		0.5	2	0	0.6	1
**G. Macrophage and liver-expressed genes**						
TLN1 (Talin 1 cytosk. memb. con.)	1971	248	74	172	159	31
ITSN1 (Intersectin 1 membran traf)	1359	0.1	0.6	0.3	0.2	1
GRN (Granulin)	2744	588	1041	1241	1443	598
BST1 (ADP-ribosyl cyclase 2)	1204	100	19	7	7	31
GDA (Guanine deaminase)	1232	0	0	0	0	0.1
HAMP (Hepsidin Iron import)	876	0.7	0.1	0	0	0
NINJ1 (Ninjurin 1 apoptosis signal?)	1122	578	323	28	25	22
HAL (Histidine amonia lyase)	1561	9	33	66	84	29
HRG (Histidine-rich glycoprotein)	0	0	0	0	0	0

## Data Availability

All the data of importance for this article are found in Table 1.
